# The Accuracy of Point-of-Care Ultrasound (POCUS) in Acute Gallbladder Disease

**DOI:** 10.3390/diagnostics13071248

**Published:** 2023-03-26

**Authors:** Bogdan-Daniel Dumbrava, Gary Alan Bass, Amro Jumean, Nuha Birido, Martin Corbally, Jorge Pereira, Alan Biloslavo, Mauro Zago, Thomas Noel Walsh

**Affiliations:** 1 Department of Surgery, Royal College of Surgeons in Ireland, Connolly Hospital, Blanchardstown, D02 YN77 Dublin, Ireland; 2 Department of Surgery, Ponderas Academic Hospital, 014142 Bucharest, Romania; 3 Division of Traumatology, Emergency Surgery & Surgical Critical Care, University of Pennsylvania, Philadelphia, PA 19104, USA; 4 Department of Surgery, Royal College of Surgeons in Ireland, Medical University of Bahrain, Busaiteen 15503, Bahrain; 5 Department of Surgery, Tondela-Viseu Hospital Center, Av. Rei Dom Duarte, 3504-509 Viseu, Portugal; 6 Department of Surgery, Cattinara University Hospital, Strada di Fiume 447, 34149 Trieste, Italy; 7 Department of Surgery, Policlinico San Pietro, Via Carlo Forlanini 15, 24036 Ponte San Pietro, Italy

**Keywords:** point-of-care, cholecystitis, cholecystectomy, ultrasonography, gallbladder

## Abstract

There is increasing recognition that point-of-care ultrasound (POCUS), performed by the clinician at the bedside, can be a natural extension of the clinical examination—the modern abdominal “stethoscope” and provides an opportunity to expedite the care pathway for patients with acute gallbladder disease. The primary aims of this study were to benchmark the accuracy of surgeon-performed POCUS in suspected acute gallbladder disease against standard radiology or pathology reports and to compare time to POCUS diagnosis with time to definitive imaging. This prospective single-arm observational cohort study was conducted in four hospitals in Ireland, Italy, and Portugal to assess the accuracy of POCUS against standard radiology in patients with suspected acute biliary disease (ClinicalTrials.govIdentifier: NCT02682368). The findings of surgeon-performed POCUS were compared with those on definitive imaging or surgery. Of 100 patients recruited, 89 were suitable for comparative analysis, comparing POCUS with radiological findings in 84 patients and with surgical/histological findings in five. The overall global accuracy of POCUS was 88.7% (95% CI, 80.3–94.4%), with a sensitivity of 94.7% (95% CI, 85.3–98.9%), a specificity of 78.1% (95% CI, 60.03–90.7%), a positive likelihood ratio (LR+) of 4.33 and negative likelihood ratio (LR) of 0.07. The mean time from POCUS to the final radiological report was 11.9 h (range 0.06–54.9). In five patients admitted directly to surgery, the mean time between POCUS and incision was 2.30 h (range 1.5–5), which was significantly shorter than the mean time to formal radiology report. Sixteen patients were discharged from the emergency department, of whom nine did not need follow-up. Our study is one of the very few to demonstrate a high concordance between surgeon-performed POCUS of patients without a priori radiologic diagnosis of gallstone disease and shows that the expedited diagnosis afforded by POCUS can be reliably leveraged to deliver earlier definitive care for patients with acute gallbladder pathology, as the general surgeon skilled in POCUS is uniquely positioned to integrate it into their bedside assessment.

## 1. Introduction

Diseases of the gallbladder are among the most frequent abdominal problems encountered in emergency departments, and over 20 million patients present annually with biliary symptoms in the United States [1]. Confirmation of gallbladder disease requires imaging, particularly ultrasound, and can be supported by diagnostic scoring [2] for timely diagnosis and management. Traditionally in Ireland and the UK, upper abdominal sonography is performed by radiology technicians and evaluated by radiologists in most centers, which may delay the diagnosis and decision-making. This is especially concerning in the environment of a busy hospital with limited resources. Point-of-care ultrasound, performed in real-time at the patient’s bedside, has the potential to minimize delay and enhance management [3]. Being non-invasive, repeatable, and easily deployable, POCUS is an invaluable diagnostic aid, particularly for surgeons. Being non-invasive, repeatable, and easily deployable, POCUS is an invaluable diagnostic aid, particularly for surgeons.

Currently, the American Board of Surgery is driving a cultural change by embedding ultrasound in surgical training and practice in several subspecialties [4]. Despite the massive growth of POCUS and its usage in so many areas, its incorporation into surgical training and into everyday practice is highly variable between continents, countries, and healthcare systems. There is also a recognized gap between usage and training [5]. Accordingly, this study adds further support to the argument for introducing POCUS into the hospital setting and integrating beginner and advanced POCUS modules into surgical training, as it can be reliably utilized to deliver earlier care for patients.

The aim of this study was to assess the accuracy of POCUS in diagnosing biliary pathology in patients with suspected gallbladder disease compared to the reference standards of radiology reports and/or surgical pathology by measuring the sensitivity, specificity, and inter-observer agreement. The secondary outcome was to compare the time to POCUS diagnosis with the time to definitive imaging.

## 2. Materials and Methods

### 2.1. Study Protocol

**Figure 1:**
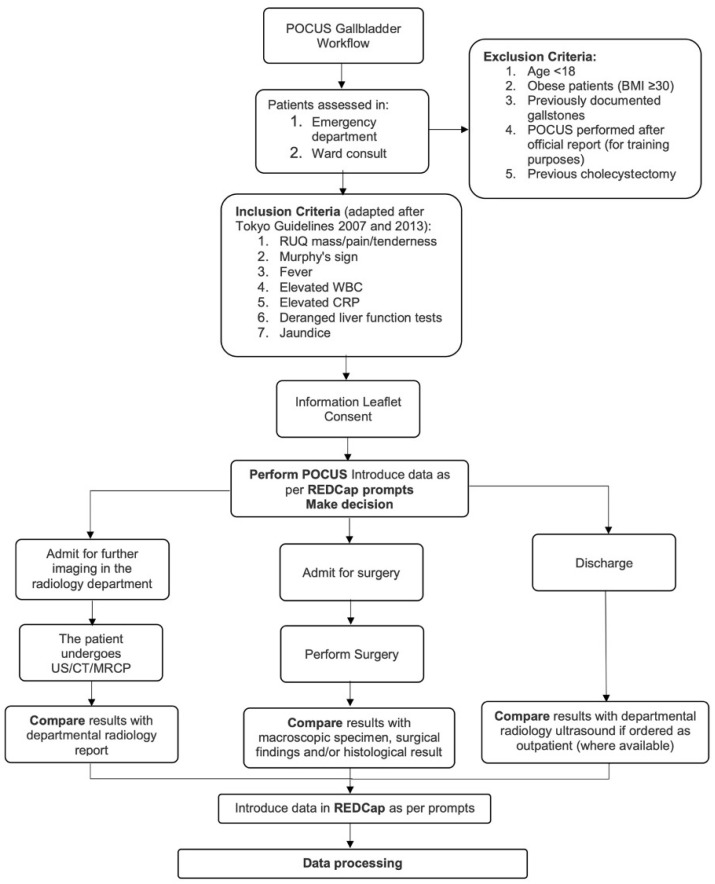
Study Workflow.

One hundred patients were prospectively recruited for this study in four international centers; Connolly Hospital, Dublin, Ireland: Tondela-Viseu Hospital Center, Portugal and Cattinara University Hospital and Policlinico San Pietro, Italy. The study protocol is presented in Figure 1.

### 2.2. Inclusion Criteria

All patients recruited to the study were 18 years of age and over and had presented with symptoms, signs and/or findings suggestive of acute biliary disease as per Tokyo guidelines [6], either in the emergency department or as a ward consultation and the availability of a POCUS trained doctor. The decision to perform POCUS was based on clinical histories, physical examinations, and laboratory investigations.

### 2.3. Exclusion Criteria

Patients with previously documented gallstone disease or patients that had previously undergone cholecystectomy were not included. Patients with a body mass index (BMI) ≥30 were also excluded.

### 2.4. Point-of-Care Ultrasound Procedure

Following clinical assessment, point-of-care sonographies were performed by four POCUS-trained surgeons (BD, JP, AB, MZ), one of whom was a surgical trainee (BD) while the other three were consultant surgeons. All four surgeons were certified tutors for the Modular Ultrasound ESTES^®^ Course [7] and had three or more years of experience in POCUS.

Sonography was performed immediately after the clinical examination without special preparation or the need for fasting. The sonographic evaluation was performed using commercially available portable scanners with a low-frequency convex probe (2.5–6 MHz) and a high-frequency linear probe (up to 13 or 15 MHz). Panoramic views were performed with the convex probe searching for free fluid, collections etc., and the linear probe, with better resolution, was used to focus specifically on the gallbladder wall to identify fluid, abscess, discontinuity, or air. Patients were examined in the supine position, with respiration held during inspiration. While assessing the gallbladder and biliary tree, they were required to turn to the lateral decubitus position.

### 2.5. POCUS Findings and Diagnosis

A proforma was completed for each patient using the REDCap^®^ platform [8]. The following data points were recorded: age, gender, hospital number, surgeon’s initials, and country. The documented sonographic findings recorded were sonographic Murphy’s sign, the presence of pericholecystic fluid, gallbladder measurements including wall thickness, the presence of gallstones or polyps, the presence of an abscess, gas in the wall, CBD diameter, or “no pathological findings”.

A negative finding was defined as a hypoechoic gallbladder without gallstones or polyps and with normal clinical and laboratory parameters.

Uncomplicated gallstone disease was defined as the detection of hyperechoic entities expressing acoustic shadowing, which were mobile with altered patient position, while gallbladder polyps resembled the hyperechoic appearance of gallstones, but without acoustic shadowing and displaceability.

A diagnosis of acute cholecystitis was made based on the combination of clinical, laboratory and the following POCUS findings: a gallbladder length >10 cm and a diameter >4 cm were considered abnormal, a positive Murphy’s sign (tenderness on pressure with the US probe), and a gallbladder wall thickness ≥3 mm [6]. Acalculous cholecystitis was diagnosed when the gallbladder exhibited the above findings in the absence of gallstones. A complicated gallbladder was defined as the presence of an abscess or free fluid around an inflamed gallbladder wall.

A common bile duct (CBD) diameter >7 mm was considered abnormal. CBD measurement was not considered a mandatory finding, and its omission was considered unlikely to lead to missing acute cholecystitis or choledocholithiasis in a routine ED presentation because of the other pathological markers that were also being gathered.

### 2.6. Clinical Decision after Surgeon POCUS

Following the POCUS assessment, patients were admitted for urgent surgery or for radiological investigations, and others were discharged for subsequent radiology or without the need for further follow-up. Following a definitive diagnosis, arrangements were made for elective surgery more than 6 weeks later when the symptoms had settled. If patients had repeated biliary colic-type symptoms and if there was no ultrasound evidence of gallstones, further imaging and clinical follow-up were arranged. Patients with unequivocally negative findings were discharged without further follow-up.

Patients admitted for inpatient radiological investigations had their imaging performed, observing local guidelines. Ultrasound examinations were carried out by radiological technicians or board-certified radiologists, while CT scans were completed by technicians and reported by consultant radiologists. All radiology consultants dictated and signed the formal reports while being blinded to the POCUS results. Patients were instructed not to reveal their POCUS findings when undergoing formal radiological examination. The time that elapsed from POCUS to the formal radiology report or surgery was recorded. Patients who were admitted for surgery without formal radiology had their macroscopic and microscopic findings compared to the POCUS examination.

### 2.7. Data Recording

POCUS findings were documented in the patients’ hospital records at the point of examination. The images were recorded with the unique hospital number for each participant, together with age and gender but without name, address, or personal information uploaded to the database. The study data were collected and managed using REDCap electronic data capture tools, and each surgeon was given a unique password with restricted access to data from other centers [7]. This ensured that the information remained anonymous and blinded in line with the General Data Protection Regulation (GDPR) (EU) 2016/679 law.

### 2.8. Ethics Approval and Consent

This prospective cohort study was approved by the Connolly Hospital Ethics Committee according to national guidelines. All patients were fully informed and provided written consent prior to entering the study. The sister institutions used this template to facilitate ethical approval in their hospitals. All patients were provided with an information pack explaining the purpose and procedural details of the study, in conformity with the guidelines of The Royal College of Radiologists and The British Medical Ultrasound Society [9,10].

### 2.9. Statistical Analysis

Analyses were done in REDCap^®^ and JamoviStats. Sensitivity, specificity, predictive values, likelihood ratios and accuracy for POCUS in detecting gallbladder content, diagnosing acute cholecystitis, and confirming the global diagnosis were calculated and compared to reference values, which were mainly the formal findings in the radiological report. Additionally, the surgical macroscopic findings were used for comparison in a few cases. The efficient-score method was used to calculate the 95% confidence intervals of the statistical calculations [11]. Cohen’s Kappa coefficient was calculated to evaluate the inter-observer agreement between POCUS and the formal radiology report, and a value of 0.81–0.99 would represent “near-perfect agreement.” McNemar’s test was used to check for a statistical difference between how often the radiologist and the surgeon endorse each other’s diagnosis, and a *p*-value of <0.05 was considered statistically significant (high concordance).

## 3. Results

### 3.1. Demographic Data

**Figure 2:**
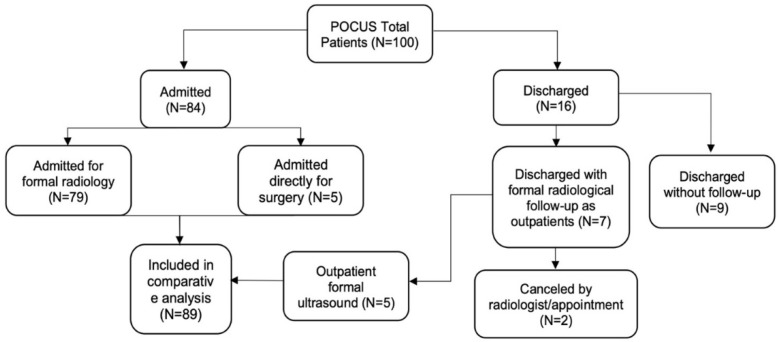
CONSORT diagram.

Over a 3-year period, a total of 100 patients were recruited to the study, of whom 66 were female, with a mean age of 50.5 (range 18–93) and underwent POCUS scanning for acute biliary pathology (Table 1). Ninety-three patients were assessed in the emergency department, and seven as inpatient consultations. Of the 100 patients recruited, 72 were recruited in Dublin, 15 in Italy and 13 in Portugal. Seventy-nine patients had formal ultrasound assessment, and five went directly to surgery and so were included in the comparative analysis. Sixteen patients were discharged after the POCUS assessment, of whom nine had no further follow-up, and seven were sent for formal radiological imaging; two of whom declined their studies (Figure 2).

### 3.2. Clinical Presentation

Ninety-six patients presented with right upper quadrant tenderness, and four others had a combination of fever and elevated inflammatory markers. A clinical Murphy’s sign was positive in 29 percent, and 15 percent had fevers. Forty-three percent of patients had an elevated C-reactive protein (CRP), 34 percent had an elevated white cell count (WCC), 27 percent had deranged liver function tests, and 11 percent had clinical jaundice (Table 1).

### 3.3. Surgeon POCUS Diagnosis and Parameters

The gallbladder was identified in all cases. The contents of the gallbladder were described in 98% of sonographies, while gallbladder measurements were recorded in 89%. Sonographic Murphy’s sign was commented on in all patients and was positive in 44% of cases. Abdominal free fluid and/or pericholecystic fluid was identified in 15% of cases. Some patients had one or more features. The CBD was identified and measured in 53% of the patients.

Of the 100 patients with suspected acute biliary disease, 36% were diagnosed with acute cholecystitis, while 26% were categorized as having symptomatic gallstones without cholecystitis. The final diagnoses for all patients are shown in Table 2. 

### 3.4. Accuracy of POCUS

Eighty-four of the 100 patients were further assessed by radiology, while five underwent surgery without prior formal radiology. The POCUS diagnosis was confirmed by radiology in 74 of the 84 (88%) patients, and the diagnosis was altered or enhanced in 10. All five patients who underwent surgical intervention had their POCUS diagnosis confirmed at surgery.

The overall global accuracy of POCUS was 88.7% (95% CI, 80.3–94.4%). The sensitivity was 94.7% (95% CI, 85.3–98.9%), the specificity was 78.1% (95% CI, 60.03–90.7%), the positive likelihood ratio (LR+) was 4.33, and the negative likelihood ratio (LR) was 0.07. There were 54 true-positive (TP) and seven false-positive (FP) diagnoses, giving a positive predictive value (PPV) of 88.5% (79.9–93.7%), while 25 true-negative (TN) and 3 false-negative (FN) diagnoses gave a negative predictive value (NPV) of 89.2% (73.18–96.2%). Excluding the five surgical cases and relying solely on radiology as the standard, the global diagnostic accuracy was 88.1% (95% CI, 79.1–94.1%) with a sensitivity of 94.2% (95% CI, 84.0–98.7%) and a specificity of 78.1% (95% CI, 60.03–90.7%). There was no statistical difference between POCUS and radiology reports (*p* = 0.342) in terms of global diagnosis. Cohen’s Kappa coefficient was 0.74, which confirms the substantial agreement between POCUS and formal radiology. Table 3 demonstrates the few areas of disagreement between POCUS and formal imaging. There were a number of cases where CT provided additional information not identified by POCUS, but this was considered outside of the scope of this study.

### 3.5. Accuracy in Diagnosing Acute Cholecystitis

Thirty-two of 84 inpatients (38%) had a clinical diagnosis and POCUS diagnosis of acute cholecystitis, of which twenty-four were categorized as acute gallstone cholecystitis, five as acute acalculous cholecystitis and three patients as complicated acute cholecystitis. Five patients underwent direct emergency surgery, and 27 of 32 (84.3%) underwent a formal radiological scan. The overall POCUS accuracy for acute cholecystitis was 92.4% (95% CI, 84.2–97.1%). Compared to radiology, the sensitivity was 88.8% (95% CI, 70.8–97.6%), and the specificity was 94.2% (95% CI, 84.05–98.8%). There was no statistical difference between the POCUS and the radiological report (*p* = 0.68). The Cohen’s Kappa coefficient was 0.92, signifying almost perfect agreement between radiology and POCUS in diagnosing acute cholecystitis. The surgical cases increased the sensitivity to 90.62% (95% CI, 74.9–98.02%).

### 3.6. Common Bile Duct Assessment

The common bile duct (CBD) diameter was measured in 53 patients, and a normal caliber was documented in 32 (60.3%), which correlated with the radiology findings. In 12 out of 53 (22.6%) patients, the CBD was considered dilated (>7 mm); 10 of these had a dilated CBD confirmed by radiology, while two were false positives.

### 3.7. The Timeframe of POCUS to Radiology or Surgery

**Figure 3:**
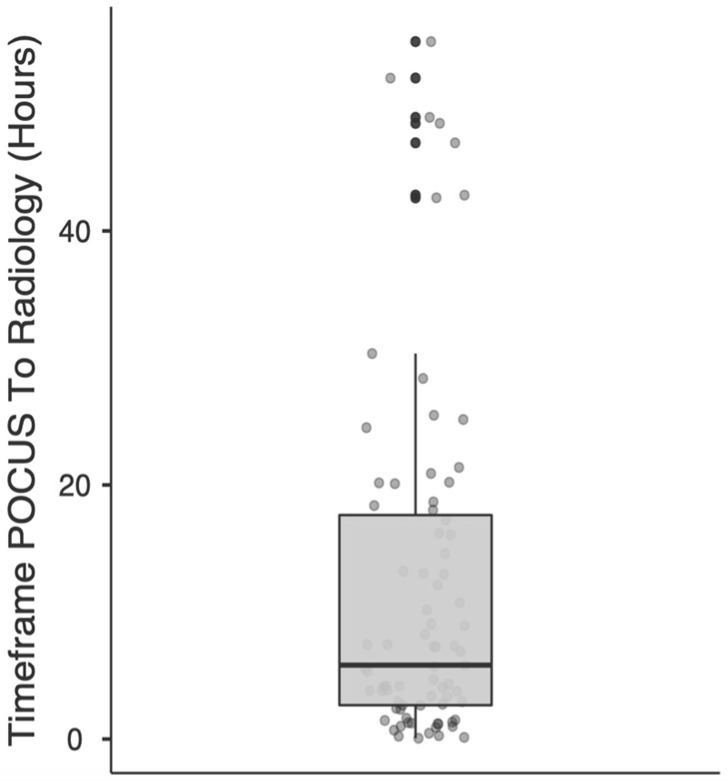
Scattergram of the timeframe of POCUS to definitive radiology report.

The interval (in hours) was calculated between the POCUS diagnosis and the time when the final radiological report was conveyed, either verbally or posted on the national integrated medical imaging system (NIMIS) for patients undergoing formal radiology (n = 79). The mean duration was 11.9 h (median 5.82), the minimum waiting time recorded was 0.06 h, and the maximum was 54.9 h (Figure 3).

In five patients, formal radiology was omitted, and the patients were admitted directly to the operating theatre. The mean time between POCUS and incision was significantly lower than the mean time to formal radiology (mean 2.30 h; median 1.62 h versus mean 11.9 h; median 5.82 h). The minimum number of hours from POCUS to the theatre was 1.5 h, and the maximum time elapsed was 5 h. Sixteen patients were discharged from the ED; nine were without follow-up as they did not have gallstones, and of the remaining seven, five had gallstones confirmed on formal ultrasound.

## 4. Discussion

This study found a high degree of accuracy for POCUS by surgeons trained in ultrasound in diagnosing acute cholecystitis and identifying the contents of the gallbladder in comparison to standard radiology. It is one of the very few studies to report on surgeon-performed POCUS diagnosis and treatment of patients without a priori radiologic diagnosis of gallstone disease [1,12]. There was a close correlation between POCUS and the radiologist’s report, with a high positive likelihood ratio. The overall sensitivity of POCUS in acute cholecystitis was 88.8% when compared to radiology alone and 90.6% when direct-to-surgery cases were included. These values are higher than reported in earlier studies [13] and even more recent surgeon-performed POCUS reports [1,14]. They are supported by the Tokyo guidelines [15] and the ESTES Consensus Statement [3]. POCUS is performed mostly on non-fasting patients, while radiologists usually insist on a 4–8 h fast before biliary ultrasound examination so that the gallbladder is distended and the stomach is empty, making gallbladder contents easier to recognize [16]. This may have influenced the false positive and false negative rates.

This study also identified multiple efficiencies derived from surgeon-performed ultrasound. Five percent of patients went directly to surgery from the emergency department without the need for further imaging. With increasing experience, these percentages are likely to improve significantly. Sixteen percent of patients were discharged from the emergency department, nine without the need for further follow-up, and four of these patients were discharged during a weekend presentation when formal ultrasound was difficult to procure. Thus, twenty-one percent of the patients had a definitive decision made at their initial clinical encounter, avoiding unnecessary delay awaiting radiology. The mean interval to the formal radiology report was 11.9 h with a maximum of 54.9 h when compared to time from POCUS to surgery with a mean duration of 2.30 h and a maximum of five hours. We have been unable to find another study that has compared timing to POCUS with timing to formal radiology.

This study affirms the value of routine clinical examinations with POCUS, performed by experienced surgeons in different European centers that share the clinical philosophy cultivated by MUSEC^®^ (Modular Ultrasound ESTES Course), with the same training, didactic protocols and sonographic diagnostic criteria [7]. Additionally, unlike most studies where emergency doctors or surgeons aimed to identify the presence of gallstones alone or identify acute cholecystitis with a limited number of secondary sonographic findings [17,18,19], our data included more advanced ultrasound features, formulating a wider range of differential diagnoses, and decreasing selection bias. The diagnosis of acute cholecystitis is challenging, even for experienced radiologists [14,15,18]. In our study, 44 of 100 patients had positive ultrasonic Murphy signs, yet the definitive diagnosis of acute cholecystitis was proven in only 32 with the aid of auxiliary features. The addition of secondary sonographic features helps rule in acute cholecystitis, while most other studies aimed at ruling out cholecystitis [1,18,19,20]. This influences the decision on whether to perform surgery, as demonstrated by Borzellino et al.; the combination of gallbladder distension, wall edema and pericholecystic fluid is highly predictive for acute cholecystitis [21]. Some surgeons have anticipated the need for conversion to open surgery based on the presence of these secondary ultrasonic findings [22].

One of the pioneering reports comparing POCUS to formal radiology was a prospective study performed by Rosen et al. [18] in the year 2000. Fifteen emergency physicians with limited or no experience in POCUS investigated 116 patients with a suspected diagnosis of cholelithiasis and acute cholecystitis. These patients would then undergo a radiology department ultrasound. The sensitivity and specificity were 92 and 78%, respectively, for detecting gallstones and 91% and 66%, respectively, for diagnosing acute cholecystitis, which is in close agreement with our study. The accuracy in detecting gallstones was in agreement with the radiology department, and thus, this study demonstrated the advantages of POCUS.

Emergency physicians [18,19,23] and surgeons [24,25] have been reporting increasing accuracy in diagnosing cholelithiasis after appropriate training. Gaspari and colleagues [26] have shown that non-radiology-trained clinicians can accurately visualize gallstones after as few as 25 focused gallbladder ultrasound examinations. Point-of-care ultrasound has, thus, been incorporated into the American College of Emergency Physicians guidelines [27]. Waiting times for clinical and radiologic diagnosis represent a bottleneck in patient management in the emergency department [28]. We believe that surgeon-performed POCUS expedites goal-directed clinical decision-making and facilitates better patient flow by ensuring rapid availability of ultrasound 24 h a day [3,29].

One of the limitations of the study is that some of the discharged patients were not followed up after the POCUS investigation. While none returned to their original hospital to report a complication or aggravation of their initial symptoms, it is possible that they presented to another hospital. Future studies should incorporate a telephone follow-up into the protocol [30]. The surgeon-sonographer was not blinded to the clinical picture, and this could be considered a bias, but equally, the patients may have inadvertently disclosed their POCUS outcome to the radiologists. The use of multiple radiologists with diverse experiences contrasts with studies where one expert specialist examiner was employed [17,22,31], which might have altered our outcomes, but we consider this a strength as it is closer to the real-world experience. A further limitation is the sample size of 100 over the 3-year period and that the cohort of patients enrolled was not consecutive, but this reflects the paucity of surgeon-sonographers. Because the study was multicentric, involving three different locations, inter-examiner agreement was not feasible, but all teams followed the same diagnostic protocol and by surgeons who were ultrasound instructors.

In conclusion, we feel that surgeon-performed ultrasound at the point-of-care is sufficiently accurate to find a regular role in the management of suspected gallbladder pathology worldwide. The identification of gallstones and secondary ultrasonic signs increases the accuracy of POCUS and indicates, with reasonable certainty, an inflamed gallbladder or its complications, facilitating early diagnosis and expediting decision-making. This study adds further support to the argument for introducing POCUS into the hospital setting and integrating beginner and advanced POCUS modules into surgical training.

## Figures and Tables

**Table 1:** Demographic Details.

Gender	
Male	34
Female	66
**Age**	
Mean age	50.5 (18–93)
**Presentation**	
RUQ pain	96 (96%)
RP elevation	43 (43%)
Leukocytosis (WCC > 11 × 109/L)	34 (34%)
Murphy’s sign positive	29 (29%)
Abnormal LFTs	27 (27%)
Fever (temperature > 37.5 °C)	15 (15%)
Jaundice	11 (11%)

**Table 2:** Final POCUS diagnoses of the patients recruited to the study.

Final POCUS Diagnosis	100 (%)
Normal findings	38 (42.7%)
Symptomatic gallstones/sludge without inflammation	23 (25.8%)
Acute gallstone cholecystitis	24 (27%)
Acalculous cholecystitis	5 (5.6%)
Complicated acute cholecystitis	3 (3.4%)
Isolated CBD dilatation	2 (2.2%)
Liver pathology	2 (2.2%)

**Table 3:** Incidence of POCUS and radiology non-agreement.

Patient Number	Explanation
1	Inconclusive diagnosis for both POCUS and radiology.
2	POCUS diagnosed gallstones. CT scan also diagnosed splenic infarction. This patient would have required CT because of atypical presentation.
3	POCUS reported no gallstones. Radiologist diagnosed a gallstone.
4	POCUS was unable to visualize the gallbladder content due to gas. Radiologist reported a difficult examination, inconclusive for acute cholecystitis.
5	POCUS diagnosed acute cholecystitis. Radiologist identified gallstones but not acute cholecystitis.
6	POCUS diagnosed acute calculous cholecystitis. Radiologist reported no acute cholecystitis.
7	POCUS diagnosed acute acalculous cholecystitis. Radiology reported no cholecystitis after 21 h of antibiotic therapy.
8	POCUS diagnosed acute cholecystitis. CT revealed a superior mesenteric artery thrombus.
9	POCUS was unable to visualize the gallbladder content due to gas. Radiologist detected gallstones after the patient had been fasting.
10	POCUS detected a gallstone in the gallbladder neck. Radiologist did not see gallstones at 48 h.

## Data Availability

The article’s data can be found as part of Dr. Bodgan Dumbrava’s thesis for a Masters of Surgery (MCh) at the Royal College of Surgeons in Ireland in 2020. https://repository.rcsi.com/articles/thesis/Point_Of_Care_Ultrasound_By_Surgeons_A_Multicentric_Trial_POCUSS_Trial_/15035529 (accessed on 8 May 2021).
